# Insufficient glutamine synthetase activity during synaptogenesis causes spatial memory impairment in adult mice

**DOI:** 10.1038/s41598-018-36619-2

**Published:** 2019-01-22

**Authors:** Hyeonwi Son, Sujeong Kim, Doo-hyuk Jung, Ji Hyeong Baek, Dong Hoon Lee, Gu Seob Roh, Sang Soo Kang, Gyeong Jae Cho, Wan Sung Choi, Dong Kun Lee, Hyun Joon Kim

**Affiliations:** 1 Department of Anatomy and Convergence Medical Sciences, Institute of Health Sciences, Bio Anti-aging Medical Research Center, Gyeongsang National University Medical School, Jinju, Republic of Korea; 2 Department of Physiology, Institute of Health Sciences, Gyeongsang National University Medical School, Jinju, Republic of Korea

## Abstract

Glutamatergic synapses constitute a major excitatory neurotransmission system and are regulated by glutamate/glutamine (Gln) cycling between neurons and astrocytes. Gln synthetase (GS) produced by astrocytes plays an important role in maintaining the cycle. However, the significance of GS during synaptogenesis has not been clarified. GS activity and expression significantly increase from postnatal day (PD) 7 to 21, and GS is expressed prior to glial fibrillary acidic protein (GFAP) and is more abundant than GFAP throughout synaptogenesis. These observations suggest that GS plays an important role in synaptogenesis. We investigated this by inhibiting GS activity in neonatal mice and assessed the consequences in adult animals. Lower expression levels of GS and GFAP were found in the CA3 region of the hippocampus but not in the CA1 region. Moreover, synaptic puncta and glutamatergic neurotransmission were also decreased in CA3. Behaviorally, mice with inhibited GS during synaptogenesis showed spatial memory-related impairment as adults. These results suggest that postnatal GS activity is important for glutamatergic synapse development in CA3.

## Introduction

Synaptogenesis refers to the creation of new synapses between neurons, and glial cells are essentially involved in these processes^1–3^. In rodents, the majority of synaptogenesis occurs by the end of the third postnatal week, and the peak takes place during the second postnatal week^4^. Specifically, most excitatory synapses in the rodent brain are formed during the second and third postnatal weeks, and this period coincides with robust astrogenesis, suggesting essential roles of astrocytes in excitatory synapse formation^5^.

Glutamate (Glu) is the major excitatory neurotransmitter in the brain, and its homeostasis is strictly controlled by Glu/glutamine (Gln) cycling between neurons and astrocytes. Glu released from neurons is taken back up from the synaptic cleft by astrocytes through excitatory amino acid transporters and then converted to Gln by the glutamine synthetase (GS). Newly synthesized Gln is transported back to the presynaptic neuron where it is converted to Glu by glutaminase. The newly synthesized Glu can take part in further glutamatergic signaling^6,7^.

Glial fibrillary acidic protein (GFAP) is the main structural protein of mature astrocytes and determines complex astrocytic morphologies, forming intermediate filament networks, contacting the blood-brain barrier, and ensheathing neuronal synapses via perisynaptic processes^8^. Growing evidence suggests that GFAP is involved in cell motility/migration, proliferation, synaptic plasticity, Glu transport, Gln synthesis, neurite outgrowth, myelination, and scar formation^9^. In rodents, each mature astrocyte occupies a specific territory consisting of 20,000–100,000 synapses^10^. In these interactions, the astrocyte processes participate in synaptic transmission and plasticity^8^. GFAP is considered an important constituent for the function and structure of astrocytes in tripartite synapses^9^.

Mature astrocytes play a major role in maintaining glutamatergic transmission homeostasis by replenishing Glu via the Glu/Gln cycle^11^. In the brain, GS is exclusively expressed in astrocytes and modulates the Gln/Gln cycle through the synthesis of Gln from Glu^7^. Alterations of GS expression and/or activity are closely linked to neurodegenerative and psychiatric diseases like Parkinson’s disease, Alzheimer’s disease, epilepsy, schizophrenia, depression, and diabetes^12–14^.

A few reports have highlighted the importance of GS activity in the neonatal period in humans and rodents. Homozygous mutations in the GS gene (GLUL) lead to abnormal brain development and severe encephalopathy^15,16^. Mice with prenatal deletion of the GS allele in astrocytes die on postnatal day (PD) 3 with low levels of Gln and GFAP in the brain^17^. However, there has been little evidence for the importance of GS activity during the synaptogenesis period for the normal glutamatergic signaling in adulthood. In the present study, we investigated the consequences of hypoactive GS during the synaptogenesis period on adult brain function, especially in the hippocampus.

## Results

### GS activity and expression significantly increase in the hippocampus from birth to the end of the third postnatal week

**Figure 1:**
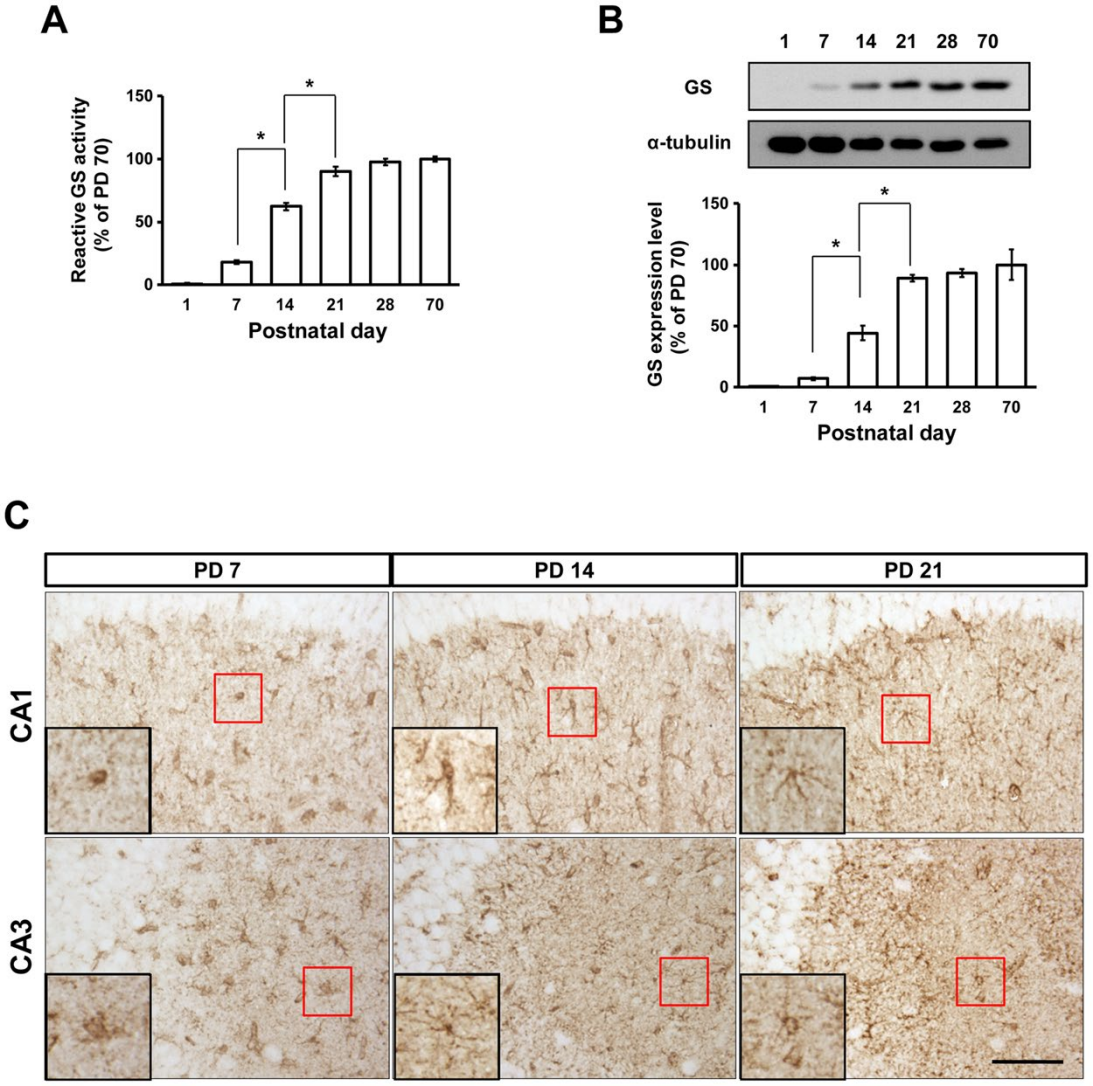
GS during postnatal development. GS activity (**A**) and expression (**B**) in the hippocampus between PD 1 and 70 (activity: n = 3 mice/group, expression: n = 3–6 mice/group). (**C**) GS immunoreactivity in the hippocampus from PD 7, 14, and 21. Scale bar: 100 μm. All values are expressed as mean ± SEM. *p < 0.05, one-way ANOVA with Newman-Keuls multiple comparison post hoc tests.

**Figure 2:**
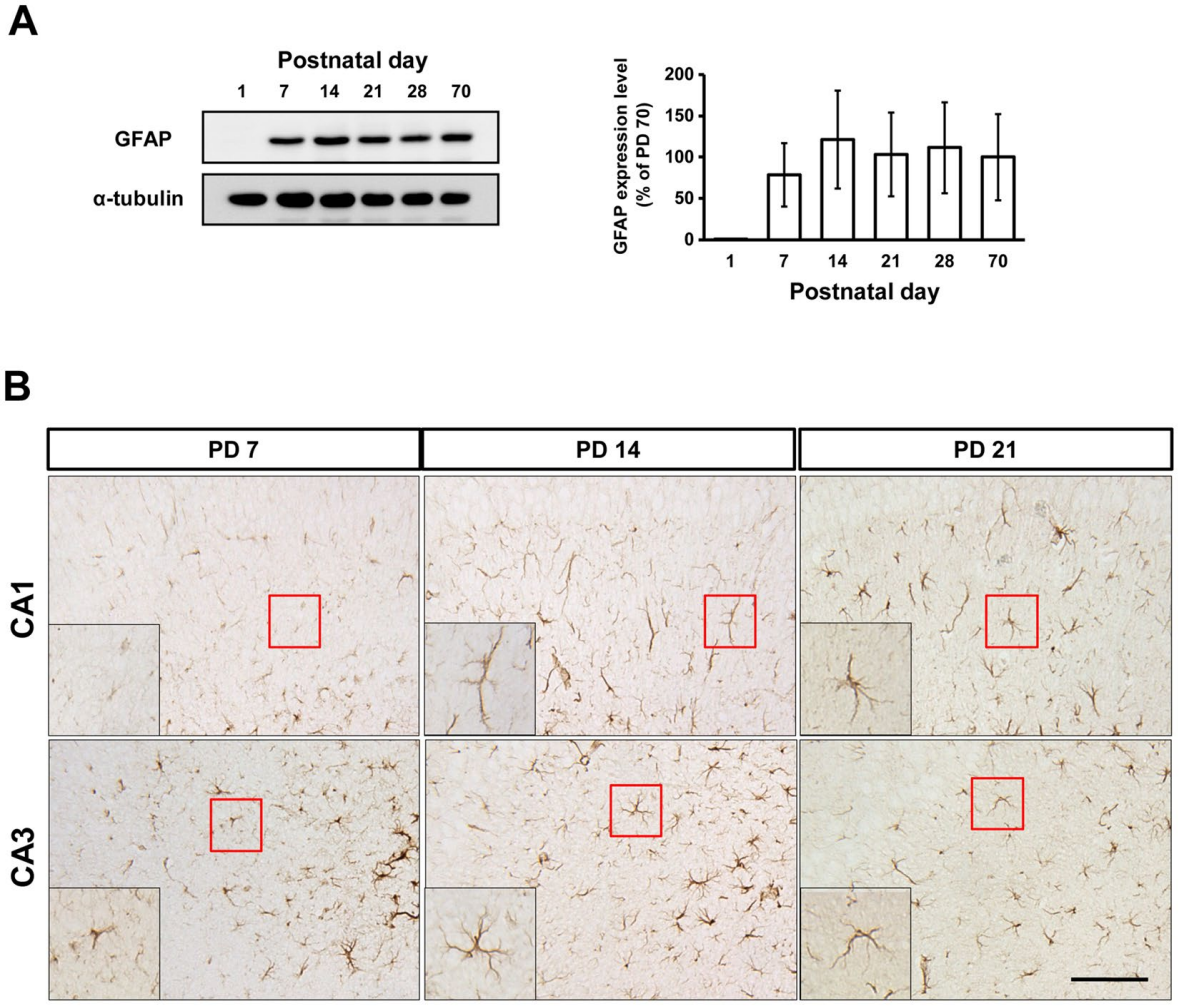
GFAP expression during postnatal development. (**A**) GFAP expression between PD 1 and 70 (n = 3 mice/group). (**B**) GFAP immunoreactivity from PD 7, 14, and 21. Scale bar: 100 μm. All values are expressed as mean ± SEM. *p < 0.05.

**Figure 3:**
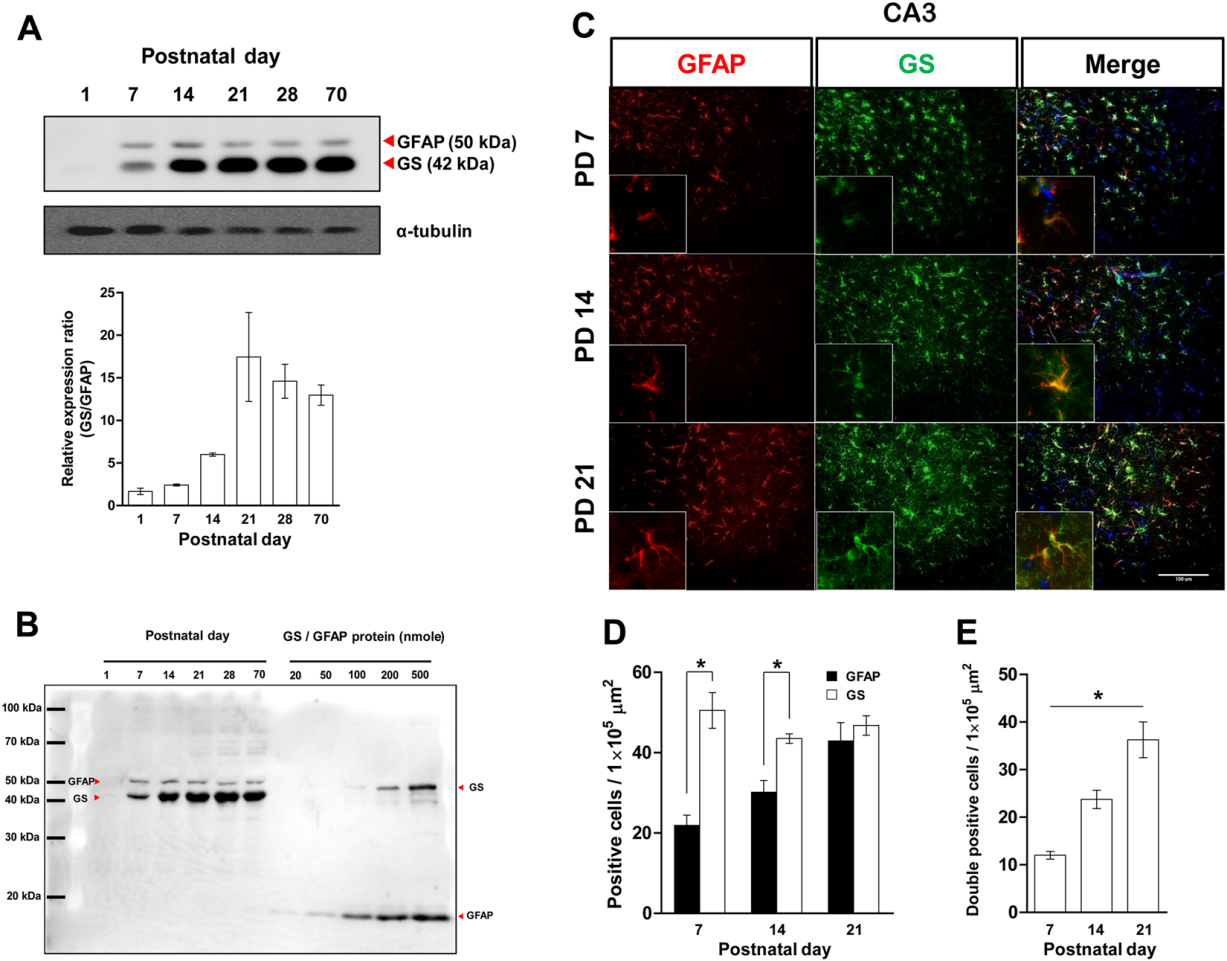
GFAP and GS expression in the hippocampus during postnatal development. (**A**) Top: GS and GFAP immunoblot. Bottom: The ratio of GS to GFAP expressions (n = 3 mice/group). (**B**) Quantitative comparison of GS and GFAP immunoblots. (**C**) Representative images of GFAP and GS immunoreactivity from PD 7, 14, and 21 in the CA3 region of the hippocampus. Scale bar: 100 μm. Single-positive cell number (**D**) and double-positive (**E**) cell number of GFAP and GS immunoreactivities (n = 4 mice/group). All values are expressed mean ± SEM. *p < 0.05, one-way ANOVA with Newman-Keuls multiple comparison post hoc tests and Student’s t-tests.

To examine the postnatal changes in GS and GFAP expression and compare these with the adult levels, we collected hippocampi from PD 1, 7, 14, 21, 28, and 70, and analyzed expression using immunoblotting and immunohistochemistry (Figs 1, 2 and 3). GS activity and expression significantly increased from PD 7 to 21 (Fig. 1A,B; activity: F_(5,17)_ = 304.8, p < 0.0001, 7 vs. 14 p < 0.05, 14 vs. 21 p < 0.05; expression: F_(5,37)_ = 23.54, p < 0.0001, 7 vs. 14 p < 0.05, 14 vs. 21 p < 0.05). After PD 21, the change in GS showed a similar plateau to the expression of synaptophysin (SYP) and postsynaptic density-95 (PSD95)^3^. GFAP expression appeared at PD 7 and did not significantly change afterward (Fig. 2A). GFAP and GS immunoreactivities were mainly found in the stratum radiatum of the CA1 and CA3 regions of the hippocampus (Figs 1C and 2B). Based on the immunoreactivities of GFAP and GS, astrocytes appeared to be cohesive and immature at PD 7 and made processes through PD 14 and 21.

### GS is predominantly expressed in the hippocampus during postnatal development

GS represents a functional link with neurons while GFAP represents a structural link with neurons^7–9^, and we found significantly increased GS during synaptogenesis with no changes in GFAP expression (Figs 1 and 2). We wanted to know the order in which these links were established, so double immunoblotting and immunohistochemistry were performed to see which protein was more abundant. Two primary antibodies against GS and GFAP were applied on one nitrocellulose (NC) membrane with different dilution rates (1/10,000 for GS and 1/2000 for GFAP). The immunoblot signal density of GS was stronger than that of GFAP, and the ratio of the GS to GFAP signals was increased by the third postnatal week (Fig. 3A). This indicated dominant expression of GS and an essential role of the protein in this critical period for normal glutamatergic synapse establishment^4^. To rule out the influence of signal intensity and affinity differences of antibodies applied, we conducted double immunoblots with the same number of molecules of recombinant proteins for GS and GFAP with hippocampal tissues on one membrane (Fig. 3B). The signal intensity of recombinant GFAP was higher than the same amount of recombinant GS, while the signal intensity of GS was stronger compared with GFAP on the same PD. Moreover, positive signals for GS in the hippocampal sections were also more abundant and showed higher intensities than GFAP at PD 7 and 14 (Fig. 3C). At PD 7 and 14, there were significantly more GS-positive cells than GFAP-positive cells, but this difference disappeared by the end of PD 21 (Fig. 3D; PD 7: t_(6)_ = 5.635, p = 0.0013; PD 14: t_(6)_ = 4.304, p = 0.0051). The number of double-positive cells significantly increased during the synaptogenesis period (Fig. 3E; F_(2,11)_ = 23.90, p = 0.0003, 7 vs. 14 p < 0.05, 14 vs. 21 p < 0.05). Collectively, these results indicate that hippocampal astrocytes express GS prior to GFAP, and GS expression predominates over that of GFAP throughout synaptogenesis.

### GS inhibition during synaptogenesis affected astrocytes and synapses in the adult hippocampus

**Figure 4:**
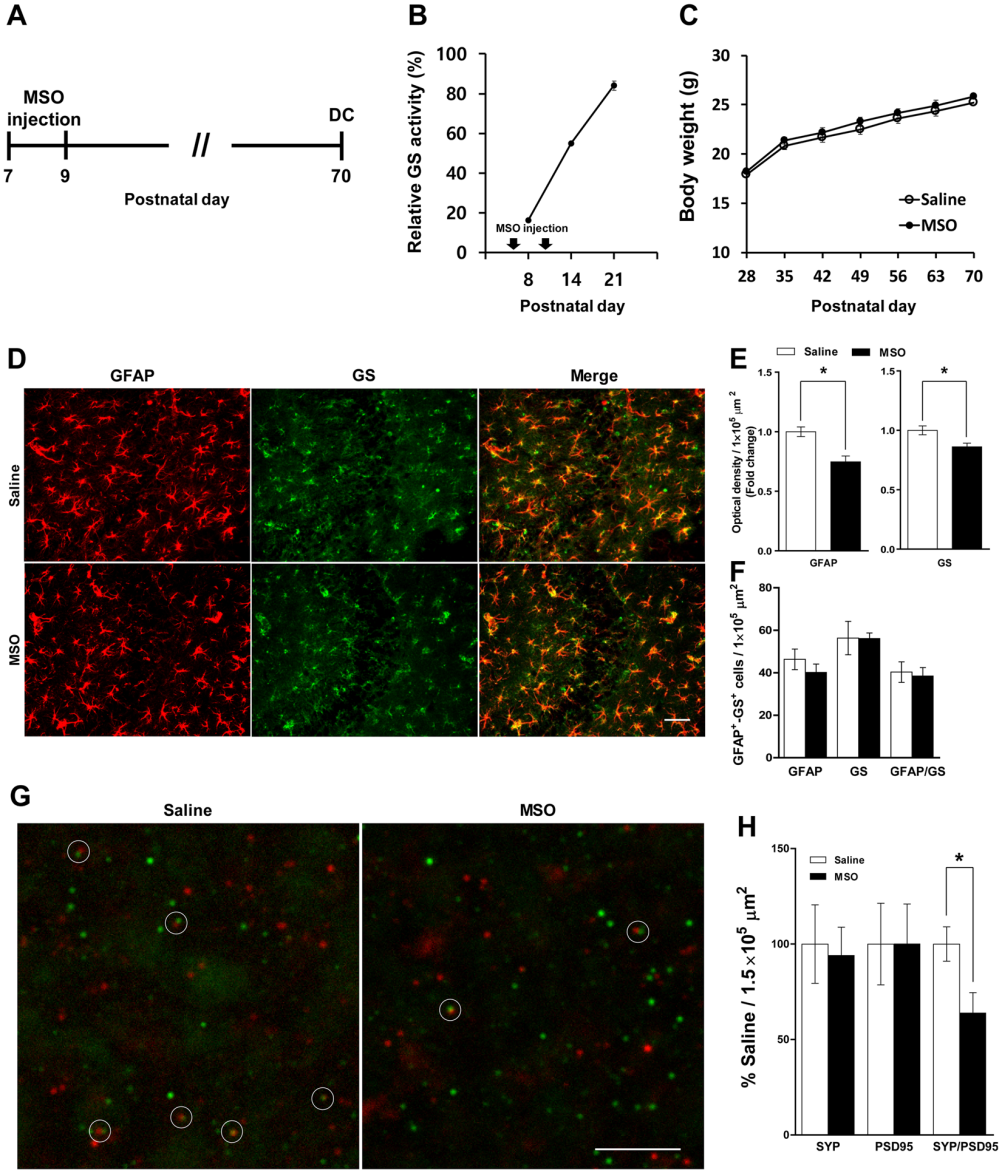
GS inhibition during early postnatal development induces structural alteration in the hippocampus. (**A**) Timeline of experiments. (**B**) Relative GS activity in the hippocampus of the saline- and MSO-injected groups from PD 8, 14, and 21 (n = 3–4 mice/group). (**C**) Body weight between PD 28 and 70 (n = 9–10 mice/group). (**D**) Representative images of GFAP and GS immunoreactivity in the CA3 of hippocampus of the saline- and MSO-injected groups from PD 70. Scale bar: 50 μm. (**E**) Fold change in optical densities of GFAP and GS immunoreactivity (normalized to saline-infused group, n = 3 mice/group). (**F**) GFAP- and GS-positive cell number (n = 3 mice/group). (**G**) Representative images of SYP (red) and PSD95 (green) in the CA3 of hippocampus of the saline- and MSO-infused groups. Scale bar: 5 μm. (**H**) Quantification of synaptic puncta (normalized to saline-infused group, n = 6 mice/group). All values are expressed as mean ± SEM. *p < 0.05, Student’s t-test. DC, decapitation.

Previous results (Figs 1–3) led to the hypothesis that synaptogenesis requires essential functional links between astrocytes and neurons through GS activity prior to structural maturation. To test the requirement for GS during synaptogenesis, we tried to see whether abnormal GS activity in this period affects adult brain function. Mice were treated with an inhibitor of GS, methionine sulfoximine (MSO, 50 mg/kg)^18^, which was injected intraperitoneally (i.p.) on PD 7 and 9 (Fig. 4A). GS activity in the hippocampus decreased to 20% of that in the saline group on the day after the first injection (Fig. 4B). Because the GS activity decrement induced by a single MSO injection recovered to the control level by PD 21 (Fig. S1), we injected twice at PD 7 and 9, which kept GS activity under 80% of the saline group by the third postnatal week (Fig. 4B). No difference was found in body weight change between the saline and MSO groups (Fig. 4C). We performed immunohistochemistry to analyze astrocytes and synaptic puncta in the adult hippocampus. The expression levels of GFAP and GS decreased in the CA3 region of the hippocampus (Fig. 4E; GFAP: t_(4)_ = 3.981, p = 0.0164; GS: t_(4)_ = 2.898, p = 0.0442), while no change was found in the CA1 region (Fig. S2). Moreover, MSO injection induced a significant loss of synaptic puncta with SYP- and PSD95-positive signals, indicating fewer functional synapses^19^ in CA3 compared with the saline group (Fig. 4G,H, t_(10)_ = 2.592, p = 0.0269).

### Hypoactive GS during synaptogenesis leads to low active glutamatergic signaling in CA3 and impaired spatial memory function

**Figure 5:**
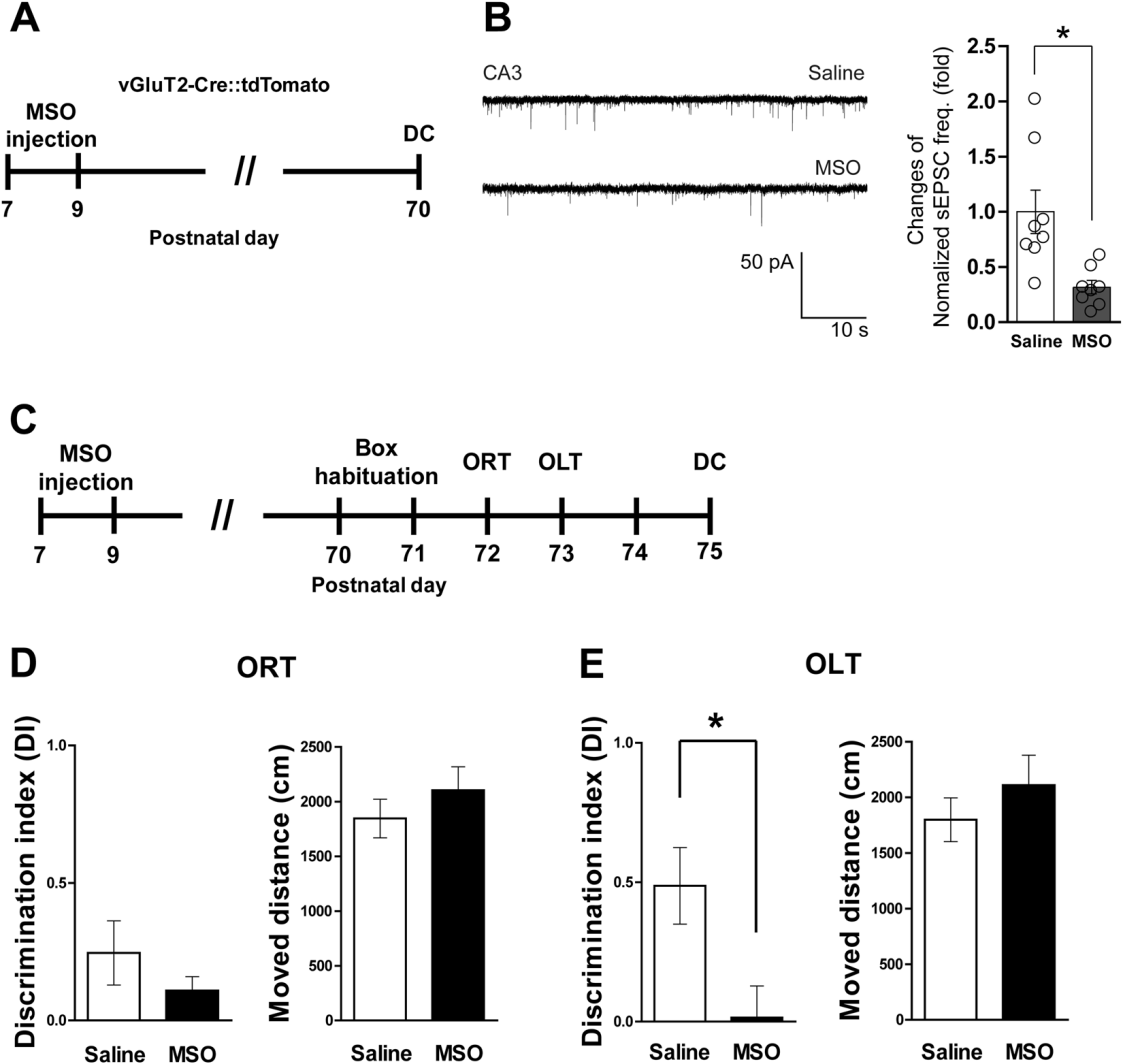
GS inhibition during early postnatal development induces hippocampus-related cognitive impairment. (**A**) Timeline of experiments. (**B**) Representative sEPSC traces in the CA3 region of hippocampus from the saline- and MSO-injected groups. The right graph shows the normalized frequency of sEPSCs (n = 8 cells/group). (**C**) Timeline of behavior tests. (**D**) DI and moved distance in ORT (n = 9–10 mice/group). (**E**) DI and moved distance in OLT (n = 9–10 mice/group). All values are expressed as mean ± SEM. *p < 0.05, Student’s t-test. DC, decapitation.

Low glutamatergic signaling would be expected based on the decreased expression of GS and GFAP in the CA3 region of the adult hippocampus (Fig. 4D,E)^20,21^. To verify glutamatergic signaling activity, we measured spontaneous excitatory postsynaptic currents (sEPSCs) of glutamatergic neurons in CA3 using vesicular Glu transporter-2 (vGluT2)-IRES-Cre::tdTomato mice and found significantly decreased sEPSC frequency in the CA3 region of the MSO-injected group compared with the saline group (Fig. 5B; t_(14)_ = 3.305, p = 0.0052). Then we tested memory function using the objective recognition test (ORT) and objective location test (OLT) (Fig. 5C). We observed a significantly decreased discrimination index (DI) of the MSO group in the OLT (t_(17)_ = 2.673, *p* = 0.0160), but there were no significant changes in the DI for the ORT or locomotor activity (Fig. 5D,E), suggesting impaired spatial memory function. These results indicate that the function of GS during synaptogenesis is essential for normal development of glutamatergic signaling and spatial memory performance of CA3-related functions in adulthood.

## Discussion

In the present study, we investigated the effect of neonatal GS inhibition during the synaptogenesis period on adult brain function using mice. GS expression and activity rapidly increased from PD 7 to 21, thus we injected MSO to inhibit GS activity to 80% that of control by the end of the third postnatal week. This resulted in decreased GS and GFAP protein expression in the CA3 region of the hippocampus at PD 70, as well as fewer synaptic puncta. Interestingly, there was a low frequency of sEPSCs on glutamatergic neurons along with impaired spatial memory function. To the best of our knowledge, this is the first report on the importance of neonatal GS activity for normal synaptogenesis in the hippocampus and adult brain functionality.

In the normal adult brain, GS is exclusively expressed in astrocytes and plays a role in neurotransmitter production and detoxification^22^. In the postnatal developing brain, GS participates in energy metabolism and glucose usage in the process of synaptogenesis^16,23^. Thus, GS activity and expression rapidly increase between the first and third weeks^24–26^, which is consistent with our results (Figs 1–3) and corresponds with the major period of gliogenesis and synaptogenesis^5^. Moreover, expression of GS was much higher than that of GFAP (Fig. 3), which is the best known astrocytic protein and has diverse roles in neural functions^9^. During normal brain development, major events such as gliogenesis and synaptogenesis must occur at specific times and the expression and activities of key regulatory molecules involved in such events are essential to accomplish these predetermined developmental programs^4^. These processes are also indispensable for normal brain structure and function in adulthood, which is supported by many previous reports^27–29^. Previous reports and our findings suggest that GS has an essential role in synaptogenesis, especially in glutamatergic synapse formation, because GS is a key enzyme in the Glu/Gln cycle^7^.

Glutamatergic synapse development requires a supply of the neurotransmitter Glu, which is supported by the cycling of Glu/Gln between neurons and astrocytes. In humans, Glu levels increase during the first year of life^30^. Similarly, in rats, Glu levels at PD 7 are half of adult values^31,32^. Thus, a substantial increase in de novo synthesis of Glu is needed during synaptogenesis, which may deplete metabolites of the tricarboxylic acid cycle. To avoid this, some metabolites must be replenished through anaplerotic processes^33^, which begin by generating new oxaloacetate through carboxylation of pyruvate via pyruvate carboxylase in the brain^34,35^. Because pyruvate carboxylase is only present in astrocytes, an anaplerotic substrate must subsequently be transferred from astrocytes to neurons^36^. This substrate is Gln, which can safely be released to the extracellular milieu without interacting with receptors. When Gln is released into the synapse, it is taken up by high-affinity transporters on neurons^37^. In the neonatal rodent brain, the amount of Gln transported from astrocytes to neurons is doubled at PD 7 compared with the adult brain and peaks at PD 14^32^. The Gln transported from astrocytes to neurons can be used as a carbon source through alpha-ketoglutarate for neuronal growth and maturation rather than excitatory neurotransmission, because there may be fewer excitatory signals until PD 12^38,39^. We inhibited GS activity until PD 21 by MSO injection, which induced Gln deficiency that disrupted the metabolic programs required for normal synaptogenesis. This neuronal Gln deficiency during maturation might result in low glutamatergic signaling activity in the CA3 region of the adult hippocampus and thus impair spatial memory function (Fig. 5), but this remains to be clarified by more specific further studies.

In addition to the metabolic needs of neuronal maturation, Gln is required for glutamatergic synapse development, which is also supported by the Glu/Gln cycle. The Glu/Gln cycle plays a key role in maintaining glutamatergic neurotransmission in the adult brain^14,21^. It begins around the first postnatal week and increases proportionately by the third week^31,40^. Transporting Gln from astrocytes to neurons coincides with the release of presynaptic Glu to persistently replenish neurotransmitters in glutamatergic synapses^11^. Here, we perturbed the Glu/Gln cycle by MSO between PD 7 and 21, which induced abnormal glutamatergic synapse establishment and resulted in hypoactive glutamatergic activity in the CA3 region of the adult hippocampus (Figs 4 and 5). This demonstrates the importance of the homeostatic Glu/Gln cycle and a sufficient supply of Gln from astrocytes for glutamatergic synapse development during synaptogenesis, which is also supported by a previous report^40^.

Although we injected MSO systemically, we only found alterations of astrocytes in CA3 (Figs 4 and S2), and this was accompanied by a low frequency of sEPSC in this region (Fig. 5). The dominant glutamatergic signaling of CA3 is due to mossy fiber pathways from the dentate gyrus^41^. The consequence of hypoactive GS during the synaptogenesis period manifested as spatial memory impairment with normal recognition memory in adulthood (Fig. 5). These findings stimulate two additional questions: (1) Why did systemically injected MSO affect only CA3? (2) Are glutamatergic synapses in CA3 or mossy fiber pathways mainly responsible for spatial memory function? Although we did not identify the detailed mechanisms for the deleterious effect of low GS activity during postnatal synaptogenesis on adult memory functions, our results suggest that normal glutamatergic signaling in the CA3 region is required for spatial memory performance. Moreover, we demonstrated the essential role of GS in the synaptogenesis period for normal brain development, especially in CA3.

## Materials and Methods

### Animals

Male C57BL/6J mice (SPF grade, KOATEC, Co. Ltd., Korea) were housed in a temperature-controlled (~22 °C) vivarium on a 12-h light–dark cycle (lights on at 6:00 AM) with *ad libitum* access to food and water. For analyses of GS and GFAP expression during postnatal development, mice were sacrificed for experiments according to their PD (1, 7, 14, 21, or 70). To assess the effects of hypoactive GS activity during synaptogenesis, mice were injected with MSO (50 mg/kg, i.p.)^18^ on PD 7 and 9. For electrophysiological experiments, we used vGluT2::tdTomato transgenic mice obtained from mating tdTomato^lox/lox^ mice (The Jackson Laboratory, USA) with vGluT2^IRES-Cre/IRES-Cre^ mice (The Jackson Laboratory) that expressed red fluorescence protein specifically within glutamatergic neurons in a Cre-dependent manner. All experimental procedures were performed in accordance with National Institutes of Health (NIH) guidelines and with a protocol (GLA-100917-M0093) approved by the Gyeongsang National University Institution Animal Care & Use Committee (GNU IACUC).

### Double immunoblotting with refined recombinant peptides

To investigate the changes of GS and GFAP expression during postnatal development, hippocampal tissues were collected from mice on PD 1, 7, 14, 21, 28, and 70. The collected tissues was homogenized in lysis buffer (#78510, Thermo Scientific, USA) with protease inhibitor cocktail (Sigma, USA) and centrifuged for 30 min at 12,000 rpm. Quantified protein was mixed with 4X sodium dodecyl sulfate sample buffer, boiled for 5 min, separated on 10% polyacrylamide gels, and subsequently transferred onto NC membrane (Whatman, USA). The membrane was blocked with 5% nonfat milk in 0.1% Tween-20/Tris-buffered saline and incubated with primary antibodies (anti-GS, 1:10,000, MAB302, Millipore; anti-GFAP, 1:2000, 18–0063, Invitrogen, USA) overnight at 4 °C. Anti-α-tubulin antibody was used as a loading control to normalize the signal intensity of each target protein. For quantitative analysis, immunoblots included quantified recombinant proteins of GS and GFAP (recombinant human GS, ProSpec, enz-544, Israel; recombinant GFAP, MyBioSource, MBS954882, USA) containing the antigenic site for each antibody.

### Assay for GS activity

GS activity assays were performed as previously described^13^. Briefly, hippocampal tissue was homogenized in 50 mM imidazole lysis buffer (pH 7.5) and centrifuged at 12,000 rpm for 30 min (4 °C). The GS biosynthetic reaction buffer contained 100 mM imidazole (pH 7.5), 50 mM MgCl_2_·6H_2_O, 100 mM monosodium Glu, 50 mM NH_4_Cl, and 10 mM ATP. Next, 90 μl of the reaction buffer and 10 μl of 30 μg crude extracts were added to PCR tubes and incubated at 37 °C for 30 min (Thermal Cycler, Bio-Rad, USA). After 30 min, 50 μl of the reaction mix was transferred to a flat-bottom 96-well plate. The color of the reaction mix was developed as described previously^42^, and the absorbance at 690 nm was measured using a microplate reader (TECAN, Switzerland).

### Immunohistochemistry

Immunohistochemistry was performed as previously described^13^. Fixed brains were sectioned (30 μm thickness, coordinates −1.34 to −2.30 mm from the bregma) and incubated with storage solution. Sections incubated with anti-GFAP (Invitrogen Corporation, 18–0063, 1:1,000), GS (Millipore, MAB302, 1:1,000), PSD95 (Abcam, ab12093, 1:500), and SYP (Abcam, ab14692, 1:500) at 4 °C overnight. Signals were visualized using an ABC kit (ABC elite Standard, Vector: PK-6100) or Alexa Fluor-conjugated secondary antibodies. Slices were mounted on gelatin-coated slides and coverslipped using Permount (Sigma) or anti-fade reagent with DAPI (Invitrogen). The single-stained images were obtained using a microscope (BX50; Olympus, Japan) equipped with a pco camera, while the double-stained images were obtained a using a DSU microscope (BX51, Olympus). We used at least three sections from each animal to count GS- and GFAP-positive cells in the hippocampus using ImageJ software (NIH, USA).

### Behavioral tests

The ORT and OLT were started on PD 70 as described previously^43,44^. Briefly, mice were habituated to a square-shaped plastic box (40 × 60 × 20 with light-grey color) illuminated at 50 lux for 10 min on days 1 and 2 (dark cycle) with visual cue and video-recording of the box from above. On day 3, two plastic shapes (<8 × 8 × 8 cm) were placed in opposite corners (7 cm from the walls) for 10 min. In the next session 24 h later, one of the plastic shapes was exchanged with a novel plastic shape (changed object “novel” and the other object remained “familiar”), and mice were exposed to this condition for 5 min. To avoid the odor cues, the objects and the box were cleaned with 70% ethanol at the end of each trial. Exploratory behavior was detected with the Ethovision XT program when the mice were sniffing or touching the object within 2 cm. Recognition memory was evaluated using the formula: DI = (time in novel − time in familiar)/(time in novel + time in familiar). After the ORT, the OLT was performed. One of the plastic shapes moved to diagonal corner from familiar location. Mice were returned to box for 5 min. Location memory was also evaluated using the DI calculated as in ORT. Before the experiment, we adjusted floor illumination and horizontality.

### Electrophysiological recording

Transverse sectioned brain slices (200 μm thickness) were prepared as described previously^45^. To record membrane current, brain slices were placed in a recording chamber superfused with artificial cerebrospinal fluid at 1.5–2 mL/min. The recording chambers were placed on the stage of an upright and infrared-differential interference contrast microscope (Olympus BX51WI), which was mounted on a Gibraltar X-Y table, and the prepared brain slices were visualized by infrared microscopy with a 40X water immersion objective. Whole-cell voltage clamp recordings were obtained from visualized glutamatergic neurons in the hippocampus at a holding potential of −70 mV. Glutamatergic sEPSCs were recorded with a multi-clamp 700B in the presence of picrotoxin (100 μM). All recordings were made at 30 ± 2 °C. The pipette solution contained the following substances (in mM): 130 KCl, 5 CaCl_2_, 10 EGTA, 10 HEPES, 2 MgATP, 0.5 Na_2_GTP, and 5 phosphocreatine.

### Statistical analysis

Data were analyzed using one-way analysis of variance (ANOVA) and Newman-Keuls post hoc tests were used for multiple comparisons. For two-group comparisons, *t*-tests were used (GraphPad Prism 5.01, USA). Data are presented as mean ± standard error. Statistical significance was set at p < 0.05.

## Electronic supplementary material

Supplementary Information

## Data Availability

All data are available upon request.
