# Supplementary material for: Insufficient glutamine synthetase activity during synaptogenesis causes spatial memory impairment in adult mice
Source: Sci Rep. 2019 Jan 22;9:252. doi: 10.1038/s41598-018-36619-2 (PMC6342969; doi:10.1038/s41598-018-36619-2)
Supplement: Supplementary file 1 — Supplementary Information [file 41598_2018_36619_MOESM1_ESM.pdf]

SUPPLEMENTARY INFORMATION

Insufficient glutamine synthetase activity during synaptogenesis causes spatial memory impairment in adult mice

Hyeonwi Son[1,†], Sujeong Kim[1,†], Doo-hyuk Jung[1], Ji Hyeong Baek[1], Dong Hoon Lee[1], Gu Seob Roh[1], Sang Soo Kang[1], Gyeong Jae Cho[1], Wan Sung Choi[1], Dong Kun Lee[2], and Hyun Joon Kim[1,*]

[1]Department of Anatomy and Convergence Medical Sciences, Institute of Health Sciences, Bio Anti-aging Medical Research Center, Gyeongsang National University Medical School, Jinju, Republic of Korea

[2]Department of Physiology, Institute of Health Sciences, Gyeongsang National University Medical School, Jinju, Republic of Korea

†The authors contributed equally to this article.

[*]**Correspondence to**:
Hyun Joon Kim, PhD
Department of Anatomy and Convergence Medical Science, Institute of Health Sciences, Bio Anti-aging Medical Research Center, Gyeongsang National University Medical School, 15 Jinju-daero 816 Beongil, Jinju, Gyeongnam, 52727, Republic of Korea
E-mail: kimhj@gnu.kr; Tel: +82-55-772-8034; Fax: +82-55-772-8039

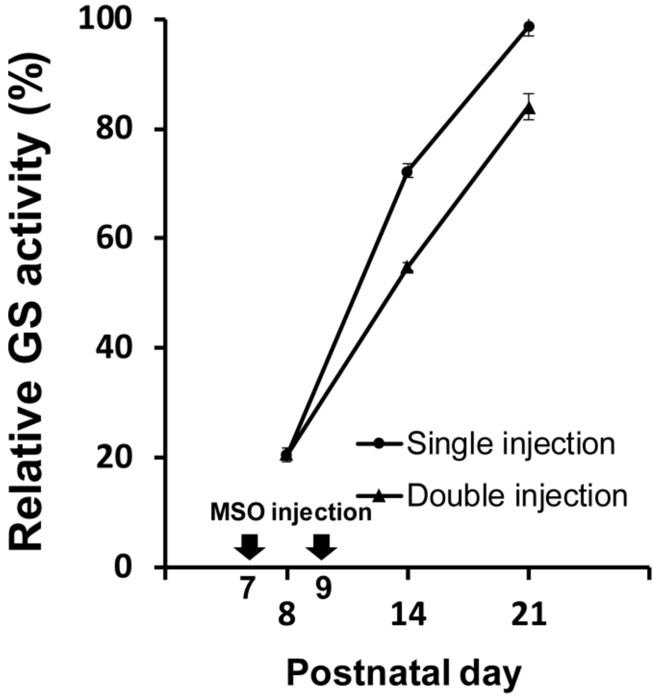

Figure S1. Hippocampal GS activity after a single or double MSO injections. All values are mean ± SEM (n = 3 mice/group).

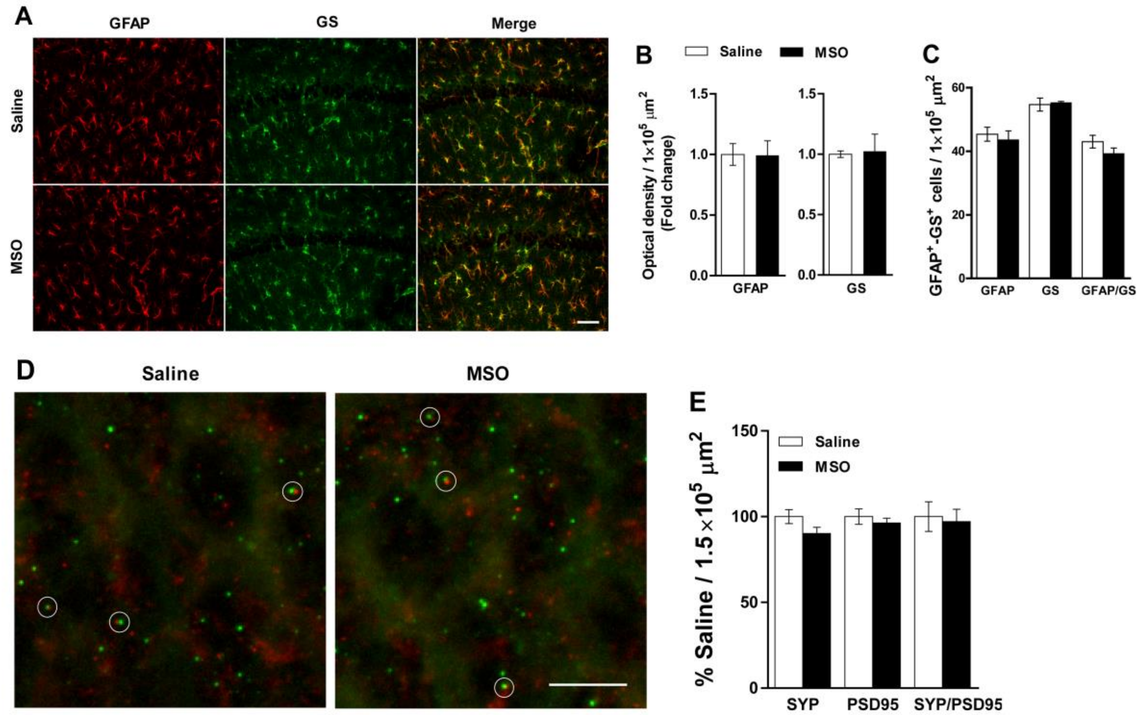

Figure S2. GS inhibition during early postnatal development did not induce structural alteration in the CA1 of hippocampus. (**A**) Representative images of GFAP and GS immunoreactivity in the CA1 of hippocampus. Scale bar: 50 μm. (**B**) Fold change in optical densities of GFAP and GS immunoreactivity (normalized to saline-infused group, n = 3 mice/group). (**C**) GFAP and GS positive cell number (n = 3 mice/group). (**D**) Representative images of SYP (red) and PSD95 (green) in the hippocampus of the saline- and MSO-infused groups. Scale bar: 5 μm. (**E**) Quantification of synaptic puncta (normalized to saline-infused group, n = 6 mice/group). All values are mean ± SEM.

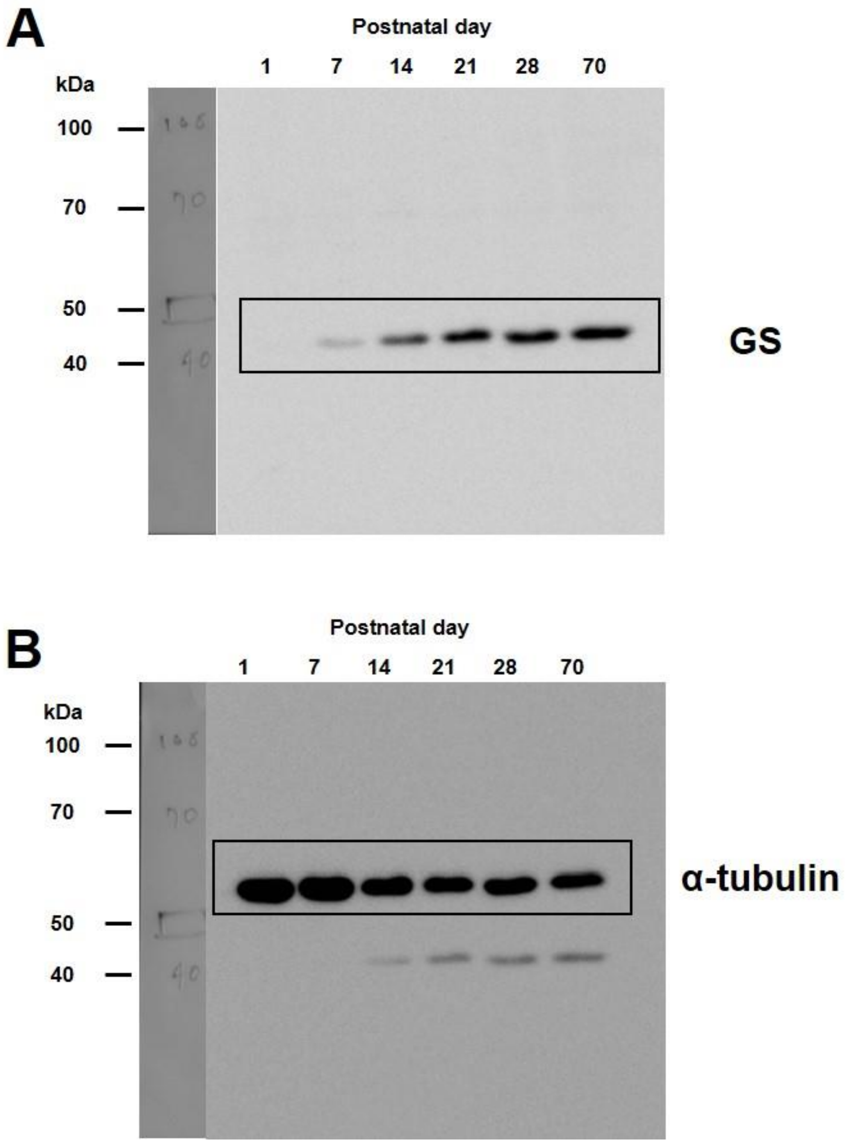

Figure S3. Uncropped western blot used in Fig 1B.

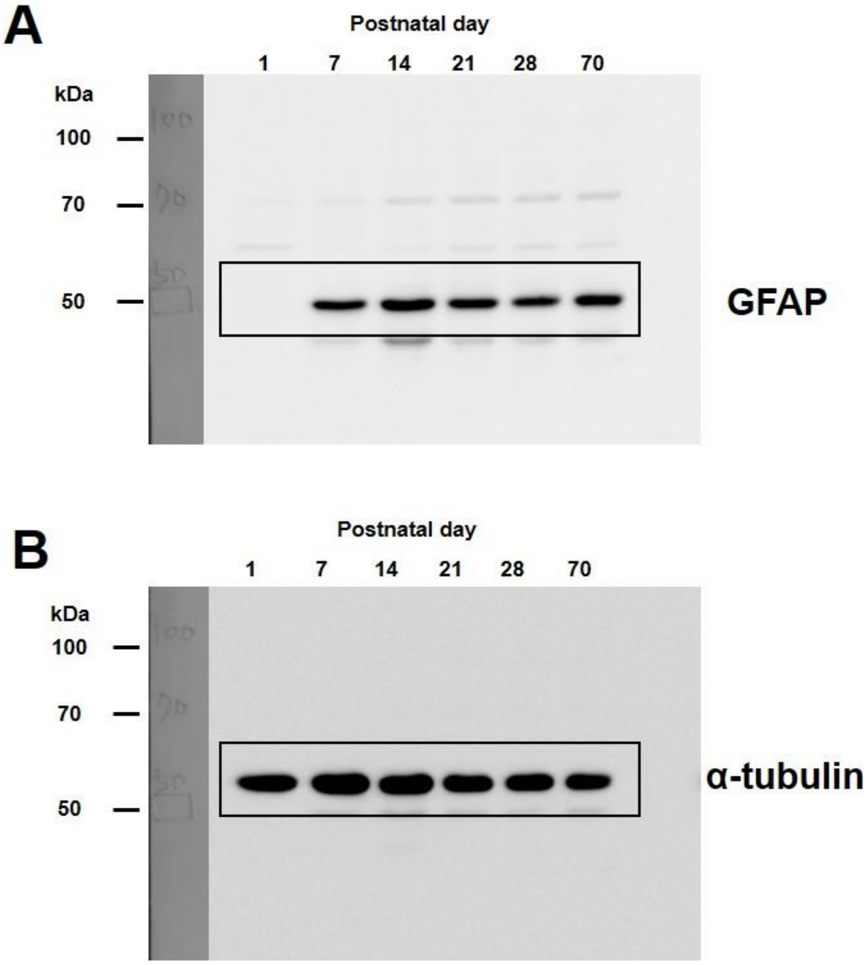

Figure S4. Uncropped western blot used in Fig 2A.

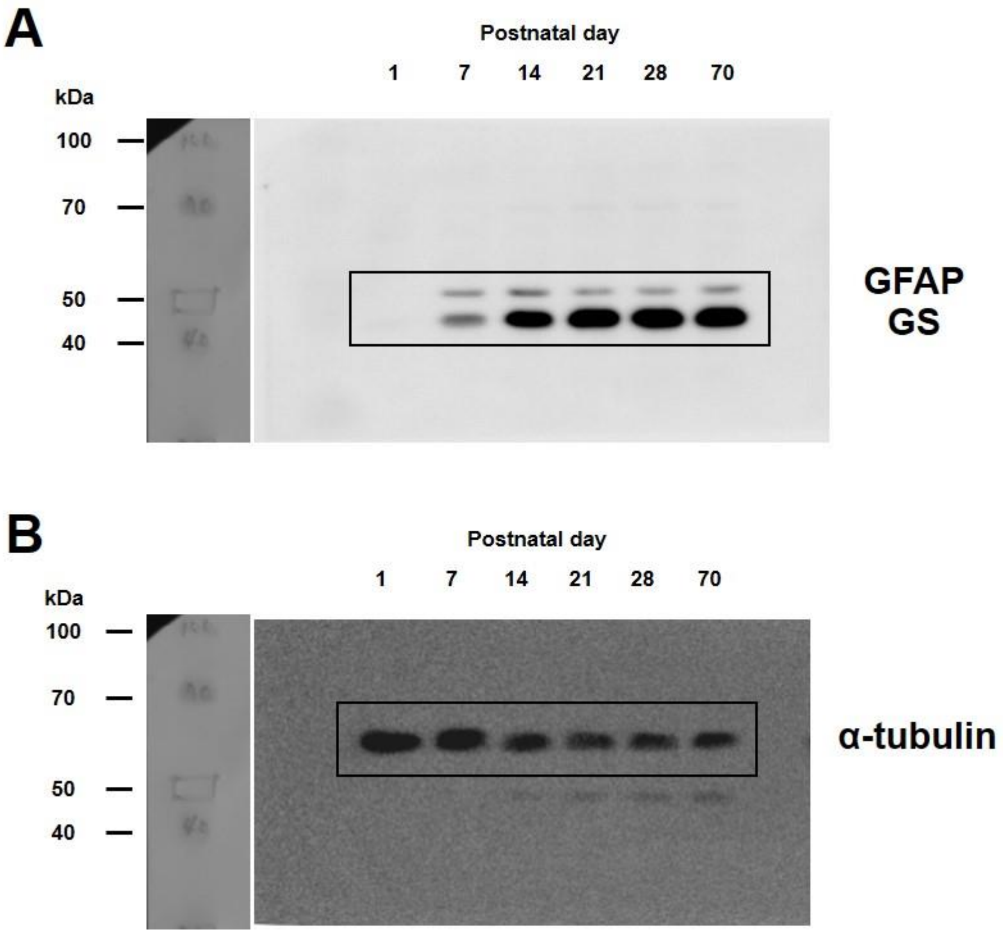

Figure S5. Uncropped western blot used in Fig 3A.

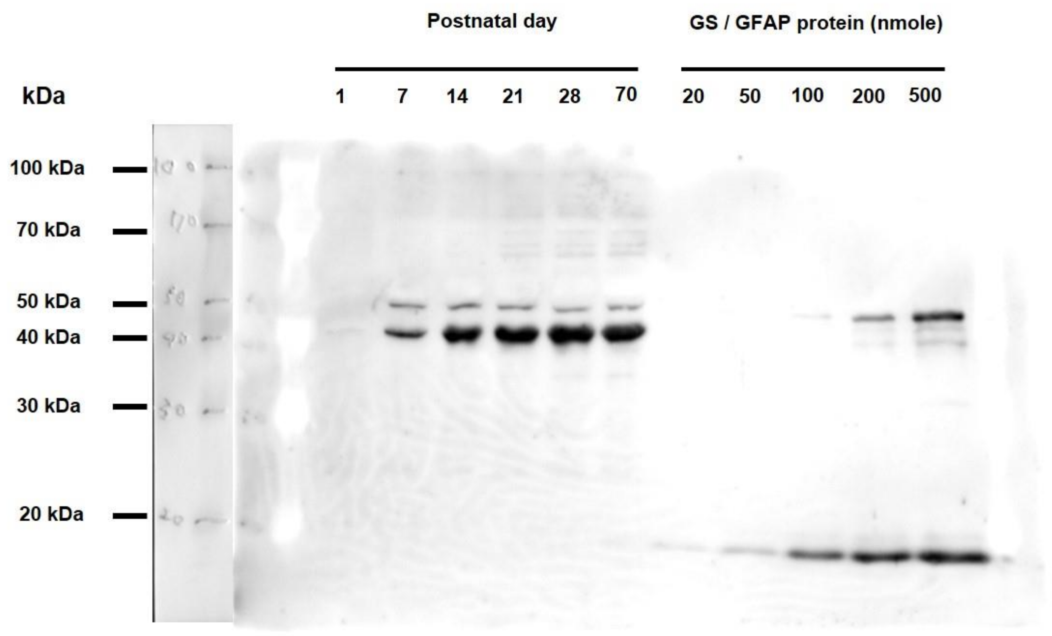

Figure S6. Uncropped western blot used in Fig 3B.
